# The Gut‒Liver Axis in Liver Disease: Molecular Mechanisms and Therapeutic Targets

**DOI:** 10.1002/mco2.70458

**Published:** 2025-11-11

**Authors:** Zhiji Chen, Siqi Liao, Suhua Wu, Siyuan Chen, Qin Tang, Li Zhou, Xiaoqin Li, Huiyi Hu, Jiayao Xu, An Zhang, Song He, Zhi‐Hang Zhou

**Affiliations:** ^1^ Department of Gastroenterology The Second Affiliated Hospital of Chongqing Medical University Chongqing China; ^2^ Department of Critical Care Medicine The Second Affiliated Hospital of Chongqing Medical University Chongqing China

**Keywords:** gut–liver axis, dysbiosis, intestinal barrier dysfunction, liver disease, pathogenesis

## Abstract

The increasing global burden of liver disease is a growing public health challenge. The gut‒liver axis, a bidirectional communication system between the intestine and liver via the portal circulation and biliary tract, is crucial for maintaining metabolic and immune homeostasis. Dysregulation of the gut‒liver axis has been recognized as a key driver of the pathogenesis of various liver diseases. However, its complex molecular mechanisms and the resulting precision therapeutic strategies remain under investigation. This review elaborates on the core components of the gut‒liver axis and the key mechanisms of gut‒liver axis imbalance in metabolic dysfunction‐associated fatty liver disease, alcohol‐associated liver disease, cirrhosis, spontaneous bacterial peritonitis, and primary liver cancer. We discuss the core roles of intestinal barrier dysfunction, dysbiosis, liver immune activation, and bacterial metabolite imbalance. Furthermore, we systematically review emerging therapeutic strategies targeting this axis, such as restoring barrier function, correcting dysbiosis, regulating bacterial metabolism, and blocking deleterious signaling. This review provides an integrative perspective on the pathophysiology of liver diseases and highlights the great potential of targeting the gut‒liver axis in translational medicine to improve the treatment paradigm for liver diseases.

## Graphical abstract

The global burden of liver disease is increasing, and gut‐liver axis dysregulation is a key driver. This article explains its mechanisms in various liver diseases and explores targeted therapeutic strategies, aiming to provide new insights into liver disease treatment. Overcoming challenges through multidisciplinary collaboration and technological innovation is key to achieving precision treatment of the gut‐liver axis.

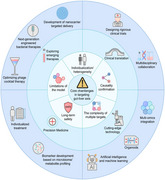

## 1 Introduction

Liver disease kills more than 2 million people each year and accounts for 4% of all deaths worldwide [1]. Currently, liver disease is the 11th leading cause of death, but liver disease‐related deaths may be underestimated [2]. Deaths are attributed mainly to cirrhosis and hepatocellular carcinoma (HCC), and the most common causes are viral hepatitis [3, 4], alcohol‐associated liver disease (ALD) [5], and metabolic dysfunction‐associated fatty liver disease (MAFLD). MAFLD is currently the most common chronic liver disease worldwide, affecting more than one‐third of adults [6], and is the fastest growing cause of primary liver cancer [7].

The gut‒liver axis is a concept that describes the complex interaction between the gut (including its microbiota) and the liver [8]. This interaction is established through the intestinal barrier, allowing substances in the gut (such as nutrients and microbial products) to be transported directly to the liver through the portal vein, while the liver regulates the gut microbiota by secreting bile and antibodies, forming a cycle [9]. Advances in research are increasingly elucidating the role of the gut‒liver axis in liver pathophysiology, solidifying its position as a key area for understanding disease mechanisms and identifying novel therapeutic targets. Despite significant progress in our understanding of the role of the gut‒liver axis in liver pathophysiology, considerable heterogeneity remains in its specific mechanisms of action across different liver diseases. Although emerging therapeutic strategies targeting this axis have shown promise in preclinical studies, their translation into clinical practice still faces significant hurdles. Therefore, it is urgent to systematically review the molecular mechanisms underlying gut‒liver axis dysfunction in major liver diseases and critically evaluate the relevant therapeutic approaches.

This review aims to comprehensively dissect the role of the gut‒liver axis in these major liver diseases. We first outline the core components of the gut‒liver axis and its communication mechanisms and then elucidate how the synergistic interactions among gut barrier impairment, microbial dysbiosis, and aberrant metabolic signaling drive disease progression. Key highlights include systematic comparisons of the involvement of the gut‒liver axis across different liver disease types to identify shared mechanisms and disease‐specific features and in‐depth exploration of key molecular pathways. The analysis further extends to emerging therapeutic strategies targeting this axis—including probiotics, prebiotics, fecal microbiota transplantation (FMT), antibiotics, gut barrier strengthening approaches, lifestyle interventions, and pharmacological modulators of metabolites or signaling pathways. Finally, this review highlights the challenges of translational medicine and explores future directions for the development of personalized gut‒liver axis interventions.

## 2 Components of the Gut‒Liver Axis and Homeostasis

The homeostasis of the gut‒liver axis is maintained by the exquisite collaboration of its core components. First, the intestinal barrier acts as a gatekeeper, defending against harmful substances through a multilayered structure. Second, the intestinal flora acts as a key signal generator, interacting with the host through its metabolites. Ultimately, the liver receives these signals through the portal venous system, and their cell populations (such as Kupffer cells and hepatocytes) integrate and process them, with the bile acid (BA) cycle acting as the core medium throughout this two‐way dialog. In this section, the functions of these components are analyzed to elucidate the molecular basis for maintaining homeostasis.

### 2.1 Intestinal Barrier: The Gatekeeper of the Gut‒Liver Axis

**FIGURE 1:**
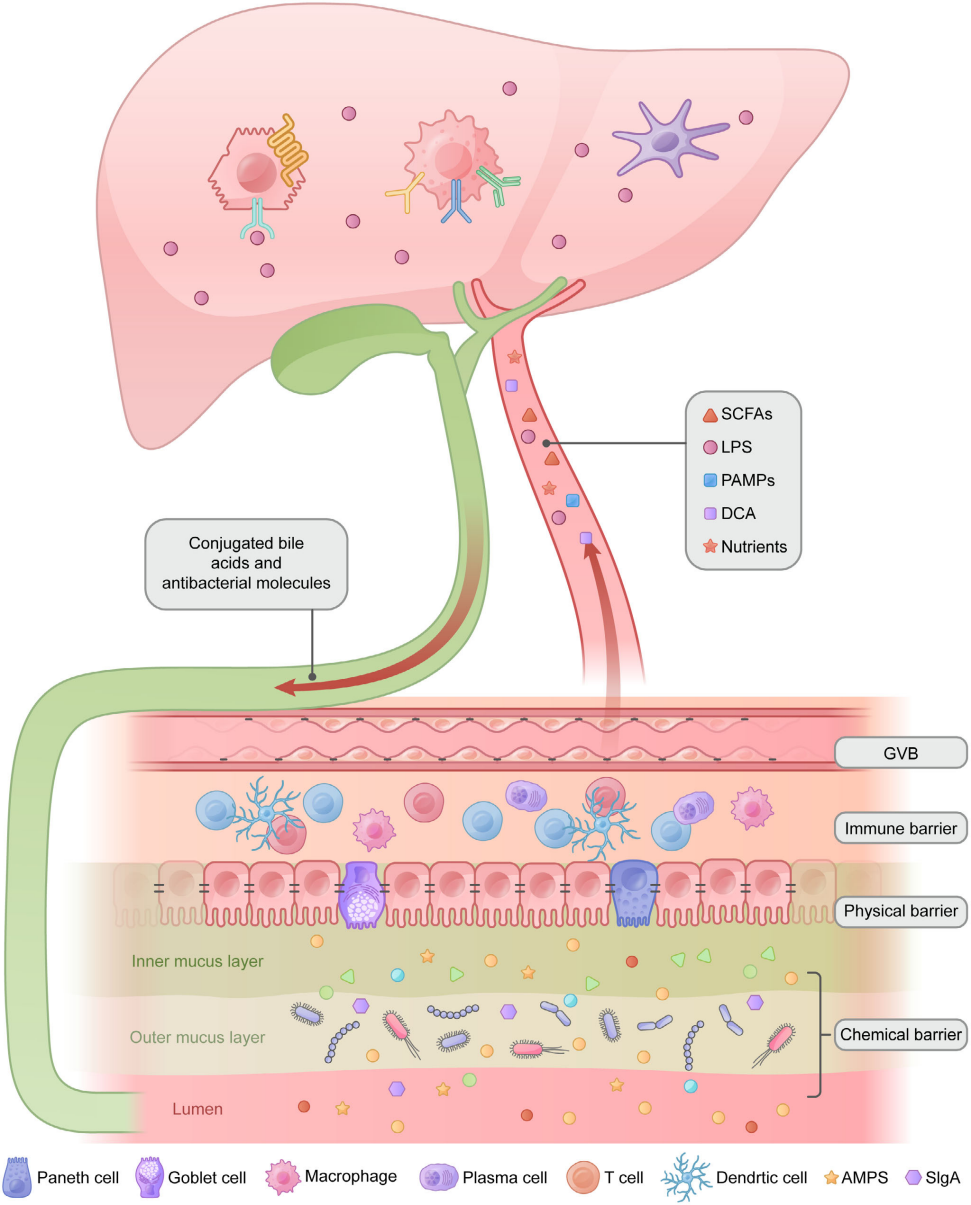
Schematic diagram of the core components and bidirectional communication of the gut–liver axis. The gut–liver axis is a concept that describes the complex interaction between the intestine (including its microbiota) and the liver. This interaction is established through the intestinal barrier, which is composed of physical barrier, chemical barrier, immune barrier, and GVB. As the first organ to receive portal blood flow from the intestine, the liver undertakes multiple functions, including detoxification, metabolism, and immune regulation. Hepatocytes, Kupffer cells, and hepatic stellate cells play a central role in recognizing and processing intestinal signals. Created with Adobe Illustrator.

The intestinal barrier isolates our bodies from environmental threats to protect our health. It comprises a physical barrier, chemical barrier, immune barrier, and gut vascular barrier (GVB) [10] (Figure 1). Intestinal barrier dysfunction is caused by intestinal microbial imbalance, intestinal permeability changes, and intestinal mucosal structure damage, resulting in the translocation of bacteria and toxic products into the blood circulation. Recent studies have revealed that increased permeability is not simply the result of disease processes but also affects disease progression.

#### 2.1.1 Physical Barrier

The physical barrier consists of the epithelium and the mucus layer. The epithelium consists of a layer of columnar epithelial cells connected by tight junctions. Columnar epithelial cells are the most numerous cell type in the intestinal epithelium. They are responsible for the absorption of nutrients and have a large number of neatly arranged microvilli to increase the absorption area. Goblet cells and Paneth cells are scattered among columnar epithelial cells. Goblet cells are responsible for the secretion of mucus, which can lubricate and protect the intestines. Paneth cells can secrete a variety of antibacterial substances, such as defensins and lysozymes, to defend against bacteria in the intestines. Tight junctions are the rate‐limiting determinants of passive paracellular transport in the epithelium [11]. They are composed of three types of transmembrane proteins, signaling molecules, and scaffolding proteins, which anchor tight junctions to the actin cytoskeleton [12, 13]. The first tight junction protein discovered was zonula occludens 1 (ZO‐1) [14], followed by ZO‐2 and ZO‐3, which are all scaffolding proteins. Transmembrane proteins include claudins, occludin, and junctional adhesion molecules. Claudins constitute the major organization component of tight junction proteins. Claudins form charge‐selective and size‐selective paracellular channels to regulate tight junction pore pathways, and the maximum diameter of the solute that can pass through them is 0.6 nm [11]. For example, claudin‐2 can mediate the passage of Na^+^ and water. Claudin‐23 regulates the permeability of paracellular ions and macromolecules by binding to claudin‐3 and claudin‐4 to redistribute them in tight junctions [15]. Occludin was the first identified tight junction transmembrane protein, but its function has not been fully elucidated. In vivo experiments revealed that mice lacking occludin can form intact tight junctions and develop normally to term. However, the measurements of mannitol flux and conductance taken in this study cannot completely rule out the possibility of barrier dysfunction [16].

The mucus layer is produced and maintained mainly by goblet cells and contains highly glycosylated glycoproteins called mucins (MUCs) [13]. The small intestine has a single, permeable mucus layer, which is nonadherent and easily penetrated by bacteria. Despite this permeable layer of mucus, unlike the dense and well‐structured mucus in the colon, bacteria cannot reach. This barrier function relies primarily on rapid intestinal transit, digestive enzymes, bile, and antimicrobial peptides (AMPs) [17]. The colonic mucus layer is thicker (approximately 50 mm in mice and 200 mm in humans [18]) and has a distinct two‐layered structure. Its core function is to keep the vast population of bacteria out of the intestinal epithelium, maintaining a peaceful coexistence. The inner layer, adhering closely to the epithelium, is essentially sterile, effectively preventing most bacteria from penetrating and coming into contact with the epithelium. The outer layer (above the inner layer) is loose and nonadherent and contains bacteria, providing a habitat and nutrient source for the intestinal commensal flora [19]. In addition to serving as a physical barrier and lubricant, mucus provides carbohydrates to some bacteria [20] and can inhibit epithelial apoptosis [21]. Both exogenous administration of the cysteine‐rich domain of Muc3 and its endogenous expression through transfection in colonic cells can reduce apoptosis induced by TNF‐α or Fas receptor stimulation, although the precise mechanisms remain unclear.

#### 2.1.2 Chemical Barrier

Chemical barriers consist mainly of secretory IgA (SIgA) and antimicrobial peptides (AMPs). SIgA is produced by plasma cells in the lamina propria, and an average of approximately 3 g per day is secreted into the intestinal lumen [22]. SIgA plays an important role in controlling intestinal infections. First, it neutralizes viral and bacterial pathogens and toxins, cutting their potential contact with intestinal epithelial cells [23]. One study [24] revealed that SIgA can clear proliferating bacteria and inhibit horizontal gene transfer by linking them together to form clumps, which is referred to as enchained growth. Additionally, SIgA can regulate bacterial protein expression and tether bacteria to mucus [23]. AMPs are small‐molecule substances secreted by Paneth cells and have inherent antibacterial activity. AMPs can be electrostatically adsorbed on the surface of bacterial membranes, easily penetrating and destroying the membrane structure, which leads to bacterial death since AMPs are usually hydrophobic [25]. AMPs include lysozyme, secretory phospholipase A2 (sPLA2), defensins, C‐type lectins of the REG3 family, and cathelicidins [26]. Lysozymes and sPLA2 kill bacteria primarily by attacking key structures of the cell wall through enzymatic activity. The remaining three AMPs kill bacteria primarily through nonenzymatic mechanisms, including the disruption of bacterial membranes and membrane potential, leading to loss of metabolites and ions, and osmotic lysis. Owing to their broad‐spectrum activity and low resistance rate, AMPs might be a promising alternative to antibiotics [27].

In the proximal small intestine, the pH, secreted enzymes, and bile also act as chemical barriers. The alkaline environment in the proximal small intestine is maintained by bicarbonate (HCO_3_^−^) from pancreatic juice and alkaline mucus secreted by the duodenal glands. This alkaline environment neutralizes gastric acid, rapidly elevating the pH of the strongly acidic chyme from the stomach to a neutral level, thereby preventing gastric acid‐induced damage to the intestinal mucosa. Additionally, alkaline pH helps regulate the gut flora by suppressing the overgrowth of acid‐dependent pathogens (e.g., *Helicobacter pylori*) while maintaining a favorable environment for beneficial bacteria. The pH and microbial density gradually increase along the gastrointestinal tract and vary significantly between individuals [28]. The digestive enzymes in the small intestine not only participate in nutrient absorption but also directly disrupt pathogenic structures. Trypsin and chymotrypsin degrade bacterial protein structures (such as pili and outer membrane proteins), whereas phospholipase A_2_ breaks down the phospholipid components of bacterial cell membranes. Bile performs multiple functions, which include maintaining the absorption of fat‐soluble vitamins, protecting the intestinal mucosa, participating in intestinal immune regulation, and promoting intestinal peristalsis. BAs stimulate MUC2 gene transcription through a protein kinase C‐dependent AP‐1 activation pathway, thereby inducing MUC production in colon cancer cells [29]. Concurrently, lithocholic acid counteracts TNF‐α‐induced degradation of intestinal barrier proteins, including ZO‐1, E‐cadherin, occludin, and claudin‐1 [30].

#### 2.1.3 Immune Barrier

When pathogenic microorganisms break through the epithelial barrier, a variety of immune cells located in the lamina propria of the mucosa take action. Mononuclear phagocytes directly process microorganisms, and plasma cells secrete antibodies into the intestinal lumen and interact with intestinal antigens [10]. Mononuclear phagocytes, including macrophages and dendritic cells, are abundant in the intestinal lamina propria [31]. Macrophages have strong phagocytic activity and can kill bacteria without external stimulation [32]. In addition, cytokines produced by intestinal macrophages are key for maintaining the activity of CD4^+^ T cells [33] and regulatory T (Treg) cells [34]. Unlike macrophages, intestinal dendritic cells absorb and transport intestinal antigens to mesenteric lymph nodes (mLNs) [35]. The mLNs form a “firewall” that prevents intestinal materials from passing through the lymphatic system and entering the blood circulation. Moreover, dendritic cells can activate group 3 innate lymphoid cells (ILC3s) via IL‐23 to produce IL‐22. IL‐22, in turn, ultimately induces Paneth cells to secrete antimicrobial molecules, such as AMPs, into the mucus layer [9]. Intraepithelial lymphocytes (IELs) are located on the basement membrane within the intestinal epithelium and interact extensively with intestinal epithelial cells. IELs comprise mainly T cells and are largely divided into induced IELs and natural IELs. These cells play key roles in maintaining intestinal barrier integrity, wound repair, and protection against pathogens [36]. Intestinal epithelial cells express specific molecules (such as HVEM proteins) to send signals to T cells, promoting their survival and patrolling [37]. The cytotoxic T cells (also called CD8^+^ T cells) in the intestinal lamina propria can directly recognize and kill intestinal epithelial cells or other cells infected by pathogens. Treg cells can inhibit excessive immune responses and prevent the occurrence and development of intestinal inflammation.

#### 2.1.4 Gut Vascular Barrier

The GVB consists of tight and adherent junction proteins between endothelial cells and the endothelia‐related enteric glial cells and pericytes. It can prevent bacterial translocation from the lamina propria into the portal circulation [9]. GVB was first identified in 2015, and enteric pathogens such as *Salmonella typhimurium* can disrupt GVB by interfering with the WNT/β‐catenin signaling pathway [38]. This is the last barrier to the entry of pathogens into systemic circulation. Enteric glial cells are involved in protecting the integrity of epithelial and endothelial barriers through the release of S‐nitrosoglutathione [39]. Compared with the blood–brain barrier, the GVB appears to be more permissive, as it allows the diffusion of larger molecules (up to 4 kDa). In this way, nutrients are allowed to pass through, but bacteria and their derived products are prevented from entering the bloodstream [40].

The main components of the intestinal barrier, which we have introduced, are also distributed differently throughout the intestine. The Brunner glands in the duodenum secrete alkaline mucus rich in HCO_3_^−^, forming a thin but rapidly renewing mucus layer. The jejunum has long, slender villi, providing a large surface area for food digestion and absorption. In addition to absorptive epithelial cells, there are mucus‐secreting goblet cells on the villi and Paneth cells that migrate to the base of the crypts. Paneth cells are characterized by dense granules rich in AMPs. The central portion of the villi consists of the lamina propria, where most intestinal immune cells are concentrated, while IELs are scattered among the epithelial cells. As the small intestine extends distally (ileum), the villi become shorter and wider, and the rate of digestion and absorption decreases. The numbers of goblet cells and Paneth cells gradually increase, whereas the number of IELs decreases. The cecum is a blind‐ended sac‐like structure responsible for the fermentation and breakdown of complex carbohydrates that are inaccessible to intestinal enzymes. The cecum lacks villi and contains only a small, flat surface epithelial area. Goblet cells are distributed throughout the crypts, while Paneth cells are rare. The colon lacks villi in all segments, and the surface epithelium primarily functions to reabsorb water from feces and provide a barrier to commensal microorganisms. Numerous goblet cells produce a dense double layer of mucus, whereas Paneth cells are extremely rare, and IELs are much less abundant than they are in the small intestine [41]. The number of gastrointestinal bacteria increases rapidly as the intestine extends. In the acidic environment of gastric juice, there are only 100–1000 bacteria per milliliter, which increases to approximately 1 × 10^5^/mL in the upper small intestine and can be as high as 1 × 10^12^/mL in the colon [42]. These region‐specific characteristics have important implications for understanding the mechanisms of gut‒liver axis diseases. Barrier defects in different intestinal segments may lead to different types of liver damage (e.g., colon barrier damage may be more likely to induce endotoxemia).

### 2.2 Intestinal Flora: Key Signal Generator

In the dynamic communication system of the gut‒liver axis, the intestinal flora and its metabolites act as core messengers and regulators, profoundly affecting metabolic homeostasis, immune balance, and disease progression in the liver. Trillions of microorganisms living in the human intestine, mainly bacteria, archaea, fungi, and viruses, which are collectively known as the intestinal flora [9, 43, 44]. Among bacteria, *Bacteroidetes* and *Firmicutes* are the main phyla in the intestinal flora, but others, such as *Actinobacteria* and *Proteobacteria*, also account for an important proportion [45, 46].

Gut bacteria play important roles in training and shaping the host's innate and adaptive immune systems, food digestion, barrier defense, and structural maintenance, modifying drug action and metabolism and producing a multitude of compounds that affect the host [47, 48]. The intestinal flora can competitively inhibit pathogen colonization, stimulate intestinal epithelial cells to secrete mucus, promote the expression of tight junction proteins, and together maintain the integrity of the intestinal epithelial barrier to prevent the translocation of harmful substances. The core function of intestinal microorganisms is to produce signaling molecules with a wide range of biological activities, such as short‐chain fatty acids (SCFAs), secondary BAs, trimethylamine, and tryptophan metabolites, which are the core mediators of the gut–liver interaction. SCFAs are a class of organic compounds produced by the fermentation of dietary fiber by the human intestinal flora. They play different roles in different physiological processes of the host and have important implications for human health and disease [49]. MUC 2 is the most important secretory MUC component in the intestine. Butyrate directly affects the production of MUC 2, and its mechanism of action may involve the selective acetylation of the MUC2 gene protein or may be indirectly mediated by fibroblasts [50]. Several in vitro cell model studies have confirmed that SCFAs (especially butyrate) upregulate the expression of intestinal tight junction proteins [49]. In mouse models, butyrate has also been shown to reduce epithelial barrier damage [51].

In addition, SCFAs play key roles in immune system regulation, affecting both innate and adaptive immune responses. Butyrate directly inhibits mammalian mTOR kinase activity, thereby enhancing macrophage phagocytosis and promoting the synthesis of AMPs. SCFAs can promote the generation of peripheral Treg cells [52] and the effector function of CD8^+^ T cell [53]. A study using a mouse model revealed that butyrate produced by intestinal Roseburia increased the efficacy of anti‐PD‐1 therapy in colorectal cancer (CRC) by activating the CD8^+^ T cell function [54]. The gut microbiome is involved in the construction of a complex signaling network through its metabolites. These molecules not only are key mediators of communication within the gut‒liver axis but also play a central role in liver metabolic regulation and immune homeostasis. These findings provide a molecular basis for developing liver disease treatment strategies based on microbiome regulation. They also suggest the need for a deeper understanding of the specific mechanisms of action of different microbiome metabolites in liver disease, which will open new avenues for future precision medicine.

### 2.3 | Liver: Receiver and Processor of Intestinal Signals

As the first organ to receive portal blood flow from the intestine, the liver performs multiple functions, such as detoxification, metabolism, and immune regulation. Hepatocytes, immune cells (such as Kupffer cells), and hepatic stellate cells (HSCs) play a core role in the recognition and processing of intestinal signals. Hepatocytes play a crucial role in receiving and processing intestinal signals, especially in recognizing bacterial lipopolysaccharide (LPS), also known as endotoxin. LPS is a major component of the cell wall of gram‐negative bacteria. It can enter the liver through the portal vein and be recognized by Toll‐like receptor 4 (TLR4) on the surface of hepatocytes [55]. As a pattern recognition receptor (PRR), TLR4 is expressed in hepatocytes and can directly sense intestinal LPS. When LPS binds to TLR4, it activates the signal transduction pathway in hepatocytes, promoting the production of a variety of proinflammatory cytokines, such as TNF‐α and IL‐6, thereby inducing an inflammatory response. Studies have shown that LPS can regulate the secretion of cytokines and chemokines by affecting multiple signaling pathways in hepatocytes and can participate in shaping the immune environment of the liver [56]. In addition, intestinal microbial metabolites can affect liver energy metabolism and lipid synthesis through specific receptors on the surface of hepatocytes. Acetate produced by *B. pseudolongum* interacts with hepatocyte GPR43 through the gut–liver axis and inhibits the IL‐6/JAK1/STAT3 pathway, ultimately inhibiting MAFLD‐associated HCC [57]. *Lactobacillus acidophilus* inhibits MAFLD–HCC by secreting valeric acid and binding to the hepatocyte surface receptor GPR41/43, inhibiting the Rho–GTPase pathway [58].

Kupffer cells are resident macrophages in the liver that play key roles in immune surveillance and regulation [59]. Kupffer cells can be distinguished from other hepatic macrophages on the basis of their location in the body and the expression of the surface molecules Tim4, Vsig4, Clec2, and Clec4f [60, 61]. They can efficiently recognize and respond to microorganisms and their metabolites from the intestine by expressing multiple PRRs, thereby initiating the body's immune response. Kupffer cells are located primarily within the bloodstream and attach to the sinusoidal endothelium [62], occupying a key position throughout the sinusoids, and their presence effectively forces pathogens of intestinal origin to pass through these highly efficient immune sentinels. When a bloodstream infection occurs, a large fraction of circulating bacteria are captured by Kupffer cells during their first passage through the liver [63, 64]. Kupffer cells can recognize pathogens via multiple PRRs, such as TLR‐2, TLR‐3, and TLR‐4 [65, 66, 67], and then coordinate immune responses by releasing TNFα, IL‐1, IL‐6, IL‐10, or other cytokines [68, 69, 70, 71].

HSCs make up approximately 5–8% of healthy liver cells and remain quiescent in the healthy liver. Following liver injury, HSCs undergo a well‐characterized activation process in which they differentiate from vitamin A‐storing lipocyte‐like cells to become the predominant fibroblast type in the liver [71, 72]. Steatotic hepatocytes, liver sinusoidal endothelial cells, and activated Kupffer cells can secrete a variety of cytokines and vesicles to activate HSCs [73]. Increased intestinal permeability and bacterial‐derived LPS can also activate HSCs [74, 75]. Activated HSCs secrete excessive amounts of extracellular matrix into the intercellular space, causing liver fibrosis and liver cirrhosis.

### 2.4 BA Circulation: The Core Medium of the Intestinal–Liver Dialog

BAs are produced from cholesterol in the liver and mediate the absorption of dietary lipids and fat‐soluble vitamins [76]. BAs can be divided into primary BAs synthesized directly by the liver and secondary BAs formed by chemical modification by intestinal bacteria [77]. Most BAs are reabsorbed in the intestine (mainly in the ileum) and transported to the liver through the portal vein system. After absorption in the liver, they are secreted into the bile duct again, resulting in enterohepatic circulation. Therefore, only approximately 5% of circulating BAs are lost in the feces, which is the main way for the body to remove excess cholesterol [78]. However, BAs are more than just digestive aids. They are also indispensable signaling molecules in the gut‒liver axis. BAs closely link the gut and liver through their unique synthesis, circulation, and receptor activation mechanisms, regulating metabolism, immunity, and microbiome balance. Studies have shown that patients with CRC have abnormally elevated levels of secondary BA deoxycholic acid (DCA), a negative regulator of intestinal CD8^+^ T cell effector function. Mechanistically, DCA inhibits Ca^2+^‐activated T cell nuclear factor 2 signaling by targeting plasma membrane Ca^2+^ ATPase, thereby suppressing CD8^+^ T cell responses [79].

Farnesoid X receptors (FXRs) belong to the nuclear hormone receptor family and are nuclear receptors for BAs. FXR is highly expressed in hepatocytes and intestinal epithelial cells (especially in the ileum) [80]. In the liver, BA activation of intrahepatic FXR induces the expression of small heterodimer partner, which forms heterodimers with nuclear receptors, inhibits CYP7A1 transcription, reduces BA synthesis, and prevents hepatotoxicity [81, 82]. In addition, FXR activation can upregulate the expression of hepatic bile salt export pump and multidrug resistance‐related protein 2, promoting the secretion of BA into the bile duct [83]. In the intestine, ileal FXR activation induces the synthesis of fibroblast growth factor 19 (FGF19), which is released into the blood and acts on the liver, synergistically inhibiting CYP7A1, and is at the core of the intestinal–liver negative feedback axis [84]. Intestinal FXR activation can also increase tight junction protein expression and reduce bacterial translocation.

Takeda G protein‐coupled receptor‐5 (TGR5) is an epithelial receptor for BAs and is significantly different from the nuclear BA receptor. The expression of TGR5 varies across different anatomical sites, with higher expression in bile duct epithelial cells, Kupffer cells, and enteroendocrine cells and lower expression in the liver [85, 86]. Katsuma et al. reported that the activation of TGR5 in intestinal enteroendocrine cells (L cells) induces the release of glucagon‐like peptide‐1 (GLP‐1). The delivery of GLP‐1 to the liver and pancreas induces insulin secretion and improves glucose tolerance in obese mice [87, 88]. TGR5 not only negatively regulates hepatic inflammatory responses by antagonizing the NF‐κB pathway in mice [89] but also plays a key role in protecting the liver from BA overload after partial hepatectomy by controlling muricholic acid and cytokine secretion [90].

## 3 Molecular Mechanisms of Gut‒Liver Axis Dysregulation in Liver Diseases

**FIGURE 2:**
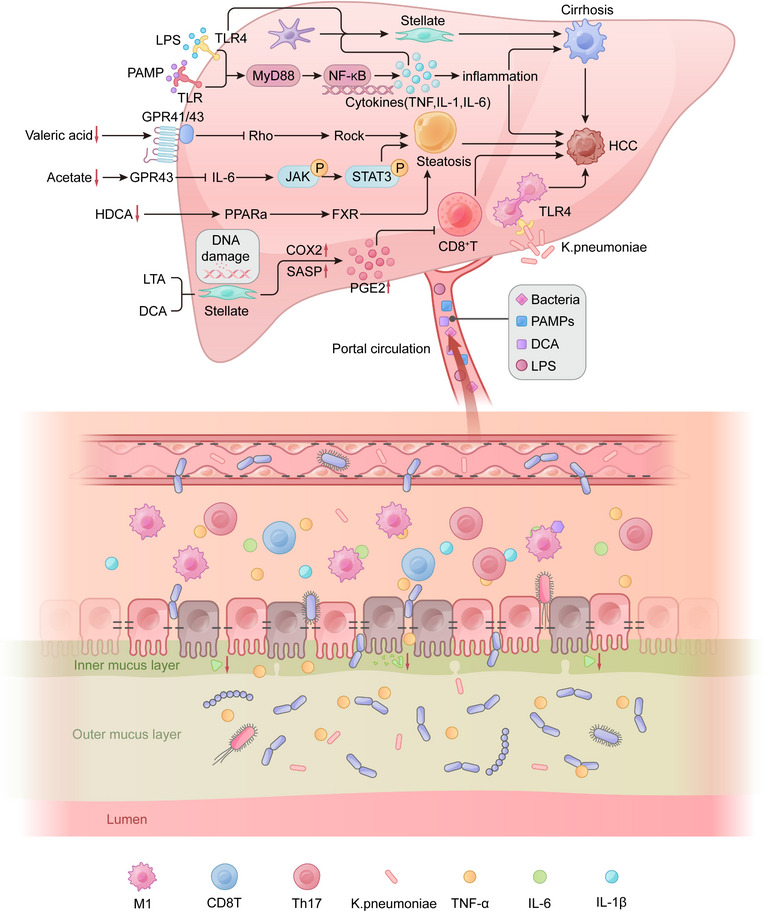
Schematic diagram of the key common molecular mechanisms driving liver disease through gut–liver axis imbalance. The primary pathogenic mechanisms of gut–liver axis‐related liver diseases include intestinal barrier dysfunction, dysbiosis, chronic inflammation, and metabolic imbalance. Claudin expression decreases, the mucus layer is disrupted, and bacteria adhere to the epithelium. Goblet cell mucus secretion and Paneth cell AMP secretion decrease, resulting in decreased antimicrobial activity. Immune cell activation (increase in M1 macrophages and Th17 cells) and CD8^+^ T cell activation decrease, leading to the release of proinflammatory cytokines (TNF‐α, IL‐6, and IL‐1β). PV‐1 expression increases, the interstitial space widens, and the endothelial barrier is damaged. Bacteria and their metabolites cross the epithelial barrier and enter the portal vein, driving hepatocyte damage, steatosis, inflammation, fibrosis activation, and abnormal proliferation. Created with Adobe Illustrator.

The pathogenesis of liver disease is an extremely complex process involving multiple factors. In this section, we discuss the main pathogenic mechanisms of liver disease related to the gut‒liver axis, including intestinal barrier dysfunction, dysbiosis, chronic inflammation, and metabolic product imbalance (Figure 2).

### 3.1 | Metabolic Dysfunction‐Associated Fatty Liver Disease

MAFLD is a definitive diagnosis based on the presence of hepatic steatosis in combination with at least one of the following three criteria: overweight or obesity, type 2 diabetes mellitus, or evidence of metabolic dysregulation. Nonalcoholic fatty liver disease (NAFLD) is a diagnosis of exclusion and is considered to be fatty liver disease caused by neither alcohol nor other factors. The definition of MAFLD directly emphasizes metabolic dysfunction as the core driver of the disease. The diagnostic criteria actively incorporate metabolic risk factors, more accurately reflecting the pathophysiology of the disease and enabling more precise prediction of disease progression, as well as hepatic and extrahepatic outcomes [91]. A meta‐analysis of 17 studies involving a total of 9,808,677 individuals revealed a high degree of overlap between MAFLD and NAFLD in the general population. However, compared with NAFLD, the new definition of MAFLD identified significantly more additional individuals than it omitted and identified more individuals with liver damage [92].

MAFLD can progress from simple hepatic steatosis (MAFL) to metabolic‐associated steatohepatitis (MASH), fibrosis, cirrhosis, and primary liver cancer. It affects more than 30% of the general population and is the fastest‐growing cause of liver cirrhosis, liver failure, and primary liver cancer [7, 93]. MAFLD patients exhibit gut dysbiosis, which manifests as a decrease in beneficial bacteria such as *Bifidobacterium* and *Eubacterium* and an increase in pathogenic bacteria such as *Escherichia coli* [94], *Klebsiella pneumoniae*, and *Veillonellaceae* [95]. Gut dysbiosis leads to the production of metabolites that can disrupt the gut barrier, ultimately causing bacteria and/or their metabolites to translocate to the liver and trigger persistent inflammation [96]. In models of high‐fat diet (HFD)‐induced MAFLD, C57BL/6J mice fed a HFD for only 1 week experienced diet‐induced dysbiosis, leading to GVB damage and bacterial translocation into the liver. This study revealed that disruption of the intestinal epithelial barrier and GVB are early events in the pathogenesis of NASH and that inhibiting GVB disruption prevents the development of NASH [97]. Few studies have explored in depth the specific mechanisms by which bacterial metabolites affect the intestinal barrier. Elevated levels of ethanolamine in the intestines of obese mice upregulate the expression of microRNA‐101a‐3p, thereby reducing the stability of tight junction protein mRNA and ultimately weakening intestinal barrier function [98]. The supplementation of a bioactive dietary fiber (glucomannan) promoted the growth of *Bacteroides ovatus* in HFD‐fed mice. Indoleacetic acid produced by *B. ovatus* is a key bioactive metabolite that enhances intestinal barrier function by activating the intestinal aryl hydrocarbon receptor, thereby improving insulin resistance [99]. A recent study [100] revealed that the portal vein area is enriched with a population of immunosuppressive macrophages that express high levels of interleukin‐10 and Marco, a scavenger receptor that can sequester proinflammatory pathogen‐associated molecular patterns (PAMPs) and damage‐associated molecular patterns, thereby inhibiting the immune response. The induction of Marco‐positive immunosuppressive macrophages is dependent on the intestinal microbiota, with commensal bacteria inducing Marco‐positive immunosuppressive macrophages to limit excessive liver inflammation. Any failure of this self‐limiting system promotes liver inflammatory diseases such as MASH.

Compared with lean people, MAFLD patients have higher rates of small intestinal bacterial overgrowth and a leaky gut, which is manifested mainly by the disruption of tight junction proteins. Furthermore, the degree of hepatic steatosis is correlated with the level of leaky gut and the presence of intestinal bacterial overgrowth [101]. The microbiota of MASH patients is enriched primarily in *Escherichia coli*, which is associated with increased endogenous ethanol production, ultimately leading to increased intestinal permeability [94]. In contrast, *Parabacteroides distasonis* and the pentadecanoic acid it produces may improve MASH by restoring intestinal barrier function and preventing the translocation of bacterial toxins [102]. Therefore, current evidence suggests that a HFD primarily leads to the destruction of GVB and other physical barriers, which in turn aggravate MAFLD.

The pathophysiology of MAFLD is complex and provides multiple potential targets for intervention, among which FXR is considered a promising target [103]. FXR is a BA‐activated nuclear transcription factor that is expressed at high levels in the liver, ileum, kidney, and adrenal gland [104]. FXR activation can affect lipid and glucose metabolism [105] and inhibit liver inflammation [106] and fibrosis [107]. Moreover, the transcription of FXR can regulate the gut microbiota [108], protect the intestinal barrier [97, 109, 110], and modulate antimicrobial activity [111]. Although obeticholic acid shows promise in reducing liver fibrosis [112], it faces multiple safety challenges and fails to achieve the primary endpoint of MASH regression. MASH response rates were similar between treated patients (11–12%) and placebo‐treated patients (8%). The most common side effects are itching and low‐density lipoprotein/high‐density lipoprotein imbalance, which may lead to adverse cardiovascular reactions [113, 114]. In addition, higher doses of obeticholic acid worsened liver function in patients with advanced liver disease and cirrhosis, and the United States Food and Drug Administration issued a “black box warning” in early 2018 [115]. Tropifexor is a selective, nonlabile acid FXR agonist [116]. In a phase II randomized controlled trial [117], compared with placebo, 12–48 weeks of tropifexor treatment resulted in significant, dose‐dependent reductions in ALT levels and hepatic fat fraction. Pruritus was the most common adverse event in all treatment groups, with a higher incidence in the 140 and 200 µg tropifexor groups. No evidence of drug‐induced liver injury was observed during the study, and some scholars currently believe that low‐dose FXR agonists combined with other drugs may play a role in the treatment of MASH [103]. MAFLD involves complex interactions among intestinal dysbiosis, intestinal barrier disruption, and liver inflammation. Future research should focus on developing novel FXR modulators with clearer targets and fewer side effects, thereby intervening in the gut‒liver axis through multiple pathways to provide effective and safe treatment strategies for MAFLD patients.

### 3.2 Alcohol‐Associated Liver Disease

ALD is among the most common liver diseases worldwide [118]. In the United States, ALD is the most common complication of cirrhosis and portal hypertension [119] and accounted for more than 40–45% of liver transplants performed in the United States in 2019 [120]. Alcohol destroys intestinal mucus, tight junction proteins, the immune barrier, and GVB mainly through its own toxic effects or through indirect effects on the intestinal flora, thereby aggravating ALD. Under long‐term alcohol consumption, some abnormal microbial metabolites may lead to intestinal barrier dysfunction. Patients with ALD have reduced levels of fecal SCFAs [121]. Feeding with ethanol (5% v/v ethanol diet for 10 days) impaired intestinal tight junction protein colocalization in female C57BL/6J mice. Preventive supplementation with tributyrin mitigated the effects of ethanol on intestinal tight junction localization disruption, intestinal permeability, and liver injury [51]. Indole is produced from l‐tryptophan by intestinal microbial enzymes, and the indole content in the stool samples of patients with alcoholic hepatitis is significantly reduced [122]. Indoles function mainly by affecting the immune barrier. The barrier in turn can stimulate IL‐22 in group 3 ILCs, thereby inducing the production of antimicrobial proteins.

Furthermore, alcohol use alters the composition and function of the gut microbiota and BA homeostasis, and these changes can be ameliorated after abstinence in patients without ALD [123]. Grander et al. [124] reported that long‐term excessive drinking led to a decrease in *Akkermansia muciniphila* levels in patients with ALD, which was indirectly correlated with the severity of liver disease. Oral administration of *Akkermansia muciniphila* to C57BL/6 mice increased intestinal mucus thickness, repaired tight junction barriers, and reduced serum LPS concentrations. In patients with established ALD, *Akkermansia muciniphila*, can be used as a therapeutic agent to improve liver damage and neutrophil infiltration. In addition to the well‐known epithelial barrier function, ethanol feeding in mice resulted in an increase in plasmalemma vesicle‐associated protein (PV1) expression, which was reduced by oral gavage of *Akkermansia muciniphila* [125]. PV1 is a marker of GVB damage. Alcohol also directly damages the intestinal barrier, and subsequent intestinal barrier dysfunction, cytokines, bacteria, and related toxins activate the immune system and promote the progression of liver inflammation and fibrosis [126].

Improving intestinal barrier dysfunction and actively reducing alcohol intake can improve patients’ liver inflammation and prognosis. Probiotics based on *Lactobacillus, Bifidobacterium, Bacteroidetes* [127], *Akkermansia muciniphila* [124], and *Micrococcus pentosaceus* [128] can beneficially affect alcohol‐induced dysbiosis and reduce liver inflammatory markers in alcohol‐fed mice and rats. One study of 46 patients with alcohol use disorder and moderate alcohol‐related hepatitis who were treated with *Lactobacillus rhamnosus* GG (LGG) or placebo revealed that 1 month of LGG treatment significantly reduced liver damage, and 6 months of LGG treatment reduced alcohol consumption to social levels [129]. A recent study [130] using metagenomic sequencing of stool samples from a multicenter, international cohort of alcoholic hepatitis patients revealed that the presence of the virulence factor KpsM, which is encoded by the *Escherichia coli* genome, was associated with patient mortality. Functional studies using germ‐free mouse models and bacterial genetic manipulations have shown that KpsM‐positive *E. coli* exacerbates ethanol‐induced liver disease and that precise targeting of the virulence factor KpsM is a promising approach to improve outcomes in patients with alcoholic hepatitis. Therefore, targeting the gut–liver axis, using probiotics as an intervention method or precisely inhibiting specific bacterial virulence factors provides a promising new strategy for the prevention and treatment of ALD.

### 3.3 Liver Cirrhosis

Cirrhosis is the terminal stage of chronic liver disease and is characterized by inflammation, necrosis, fibrosis, and regenerative nodule formation [131]. Cirrhosis is an important cause of death in patients with chronic liver disease worldwide [132] and was associated with 2.4% of global deaths in 2019 [133, 134]. China is a country with a large hepatitis B population and a heavy burden of liver disease. According to estimates from the Polaris International Epidemiology Collaboration, the prevalence of HBsAg in the general population in China was 6.1% in 2016, and there were 86 million cases of chronic HBV infection [135]. HBV infection has decreased greatly because of systemic preventive efforts, but the prevalence of HCV infection continues to increase. Moreover, the incidence of obesity and alcohol consumption increase year by year. Thus, the epidemiology and burden of cirrhosis are changing [133, 136, 137].

Previous theories suggest that intestinal barrier dysfunction is a late manifestation and complication of liver cirrhosis, but increasing evidence has shown that it appears earlier, aggravates liver inflammation, and promotes hepatic fibrogenesis. The severity of cirrhosis is associated with the levels of endotoxemia and systemic inflammation [138]. A previous study revealed that fecal microbial and metabolite profiles can independently predict MAFLD‐related cirrhosis [139]. Cirrhosis is associated with increased abundances of *Escherichia coli*, *Klebsiella pneumoniae*, and *Veillonella parvula* and decreased abundances of *Eubacterium eligens*, *Eubacterium rectale*, and *Faecalibacterium prausnitzii*. A cross‐sectional study revealed that the LPS level and percentage of *Escherichia coli* DNA‐positive patients (88 vs. 3%, *p* < 0.001) in the serum of patients with cirrhosis were significantly greater than those in the control group [140]. Endotoxin‐driven TLR4 activation mediates TGF‐beta signal transduction and HSC activation, thus constituting an important mechanism of advanced cirrhosis [74]. Similarly, mucosal barrier failure and GVB disruption occur early in MAFLD and ALD and worsen as the disease progresses to cirrhosis [97, 110, 141]. Previous studies [110, 142] have shown that the intestinal tissue of rats and humans with cirrhosis has reduced mucus layer thickness and fewer goblet cells. Prehepatic portal hypertension caused by partial portal vein ligation (PPVL) did not damage the intestinal barrier, and only CCl4‐induced cirrhosis rats exhibited reduced mucus layer thickness and goblet cells and decreased expression of ZO‐1, occludin, and claudin. GVB was destroyed in cirrhosis rats, resulting in ileal extravasation of large 150 kDa‐FITC–dextran [110]. Another study suggested that the antibacterial activity of Paneth cells were significantly lower in the ileum and cecum in animal models of cirrhosis than in models of portal vein ligation [143]. Decreased numbers of ileal goblet cells [110], reduced ileal and colonic mucus thickness [142], decreased intestinal SIgA [144] and AMP levels [143], decreased small intestinal and colonic tight junction protein levels [142], and GVB damage [97], all contribute to impaired intestinal barrier function. All these manifestations can be observed in patients with cirrhosis and in animal models. As chronic liver disease progresses to cirrhosis, live bacteria and PAMPs migrate from the intestinal lumen to mLNs or portal circulation, escaping clearance by the liver immune system and aggravating systemic inflammatory responses, immune dysfunction and complications of cirrhosis [10, 138]. A vicious cycle is formed, including intestinal dysbiosis, increased intestinal permeability, deterioration of liver function, systemic inflammation and infection, ultimately leading to poor prognosis. Therefore, interventions targeting the gut‒liver axis, such as regulating dysbiosis, repairing the intestinal barrier, and reducing bacterial translocation, have become important therapeutic strategies to interrupt the vicious cycle of cirrhosis and improve patient prognosis.

### 3.4 Primary Liver Cancer

Primary liver cancer is the leading cause of death in patients with compensated cirrhosis and the third leading cause of cancer‐related death worldwide [145, 146, 147, 148]. Seventy‐five percent of liver cancer cases occur in Asia, with China having the greatest number of cases because of its high incidence rate and large population [149, 150]. Excessive extracellular matrix deposition and increased stiffness are typical features of solid tumors such as HCC [151]. Primary liver cancer is increasingly associated with hepatitis C, treatment‐suppressed hepatitis B virus, heavy alcohol consumption, and MAFLD as major risk factors [152, 153]. Disruption of the intestinal barrier can facilitate the translocation of intestinal bacteria or related metabolites, thus impacting liver cancer development. A recent study [154] demonstrated that the transplantation of fecal samples from patients with HCC to wild‐type mice resulted in intestinal barrier impairment and the translocation of viable bacteria to the liver, where they spontaneously induced liver inflammation, fibrosis, and dysplasia and accelerated disease progression in a mouse model of HCC. Metagenomic analysis and bacterial culture of liver tissue revealed enrichment of the enteric pathogen *Klebsiella pneumoniae* in both HCC patients and mice transplanted with the HCC microbiota. The *Klebsiella pneumoniae* surface protein PBP1B interacts with and activates TLR4 on HCC cells, leading to increased cell proliferation and activation of oncogenic signaling. In addition, the intestinal flora and its metabolites also affect the tumor immune response in patients with HCC, including macrophages [155], Tregs [156], B cells [157], CD8^+^ T cells [158], and CD4^+^ T cells [159]. Immune checkpoint inhibitors have opened up promising new avenues for the treatment of HCC. However, response rates vary, and many patients fail to achieve significant benefits from these therapies. Strategies that modulate the gut microbiota offer a potential approach to increasing the efficacy of immunotherapy for liver cancer [160, 161].

In recent years, the intratumoral microbiota has attracted extensive attention in various types of tumors [162, 163, 164]. The intratumoral microbiota is considered a component of the tumor microenvironment (TME) and has been reported to regulate the tumorigenesis, progression, and prognosis of gastrointestinal tumors [162, 165, 166]. One study included 70 specimens, including 12 chronic hepatitis B tissues and 29 pairs of tumors and corresponding adjacent HCC tissues, and revealed that the heterogeneity of the microbiome in HBV‐related HCC tumors was associated with differences in clinicopathological characteristics and the TME. The heterogeneity of the tumor microbiome may serve as a biomarker for predicting the prognosis of patients with HCC [167]. Previous studies have suggested that the metalloprotease gelatinase E (GelE) from *Enterococcus faecalis* can damage the epithelial barrier and cause intestinal inflammation [168]. A comparison of HCC patients with cirrhosis patients without HCC revealed that the abundance of *Enterococcus faecalis* in the feces of patients with HCC was significantly increased [9, 169]. Another study revealed that GelE‐positive *Enterococcus faecalis* increased intestinal permeability and subsequently colonized the liver to promote the occurrence of liver cancer [170]. An imbalanced gut microbiota leads to increased bacterial abundance in liver tissue from patients with cirrhosis, ultimately resulting in significant transcriptional changes, including the activation of fibroinflammatory pathways and circuits that mediate cancer immunosuppression [171]. An antibiotic cocktail (ABX, consisting of vancomycin, neomycin, and primaxin) can inhibit liver tumors by altering the abundance of gut bacteria, as shown in a study using a primary liver model and three liver metastasis models [172]. *Bifidobacterium longum* was found to enhance liver function recovery in patients after HCC surgery, and oral administration of a probiotic mixture containing *Bifidobacterium longum* reduced the rate of delayed recovery, shortened the hospital stay, and increased the 1‐year overall survival rate [173].

BAs in microbial metabolites can act as messengers to regulate the level of the chemokine CXCL16 on liver sinusoidal endothelial cells, thereby controlling the accumulation of CXCR6^+^ hepatic NK/T cells. The accumulated NK/T cells can inhibit liver tumor growth. Secondary BAs reduce CXCL16 expression, whereas primary BAs have the opposite effect. Depletion of bacteria that mediate primary BA conversion with vancomycin is sufficient to induce hepatic NK/T cell accumulation and reduce liver tumor growth [172]. In addition to directly affecting the immune function of the liver, secondary BAs can also increase the colonization of the intestine by GelE‐positive Enterobacter faecalis, thereby damaging the intestinal barrier and promoting the occurrence of liver cancer [170]. Valeric acid derived from *Lactobacillus acidophilus* can inhibit the occurrence of NAFLD‐related HCC by inactivating the oncogenic Rho–GTPase signaling pathway [58]. *Bifidobacterium pseudolongum* can repair the intestinal tight junction barrier and inhibit the IL‐6/JAK/STAT3 pathway by producing acetate, ultimately inhibiting the occurrence of liver cancer [57]. Butyrate levels can be significantly increased by *Bifidobacterium longum*, thereby repairing the intestinal barrier, reducing bacterial translocation, and promoting recovery after liver cancer surgery [173].

Cholangiocarcinoma (CCA) is a malignant tumor of epithelial cells that originates from different parts of the bile duct system and accounts for 10–15% of primary malignant liver cancers, and its incidence has been increasing in recent years [174, 175]. Furthermore, 16S rRNA sequencing of 99 intrahepatic CCA (ICC) tissues revealed high abundances of *Burkholderiales, Pseudomonadales, Xanthomonadales, Bacillales*, and *Clostridiales*. *Paraburkholderia fungorum* was significantly more abundant in adjacent tissues and was negatively correlated with CA199 levels. Further mechanistic investigations revealed that *Paraburkholderia fungorum* may inhibit tumor growth by regulating alanine, aspartate, and glutamate metabolism [176]. The consistency of bacteria represented by *enterococci* and *staphylococci* in bile and tissues within CCA suggests a potential microbiota switching pathway from the intestine via bile to tissues within CCA [177].

The intestinal barrier also affects metastatic liver cancer. Bertocchi et al. [178] reported that in patients with CRC, the virulence factor VirF from intestinal tumor‐resident *Escherichia coli* disrupts GVB, promoting bacterial translocation and the formation of a premetastatic niche in the liver. PV‐1, an indicator of GVB destruction, is a marker of distant recurrence and liver metastasis of CRC. These findings not only confirm the key role of gut‒liver axis dysregulation in liver cancer development but also provide a theoretical basis for the development of novel liver cancer prevention and treatment strategies based on microbiome regulation. However, current research focuses primarily on preclinical studies, and future studies will need to further validate the therapeutic effects of gut‒liver axis interventions in clinical studies.

### 3.5 Spontaneous Bacterial Peritonitis

Spontaneous bacterial peritonitis (SBP) is an infection of ascites in the absence of an obvious intra‐abdominal source of infection, such as intestinal perforation or intestinal abscess [179]. It is among the most common complications in liver cirrhosis patients. According to a recent European study, the prevalence of SBP among hospitalized patients is 11.3% [180]. In a prospective study of 1302 patients with cirrhosis, SBP (27%) was the most common infection, followed by urinary tract infection (22%), pneumonia (19%), and skin/soft tissue infection (8%) [181]. Despite improvements in treatment, it is still associated with mortality rates of up to 30% and high rates of recurrence after initial infection [182]. Bacterial infection should be suspected when patients with cirrhosis experience worsening, especially if encephalopathy, acute kidney injury, or jaundice develop. The diagnosis of SBP is based on an ascites absolute polymorphonuclear leukocyte count greater than 250 cells/mm^3^ [183]. It is important to isolate a microorganism from ascites or blood so that antibiotic susceptibility results can guide antibiotic treatment.

The pathophysiological process of SBP remains largely unclear. Changes in intestinal microorganisms, alterations in intestinal permeability, bacterial translocation, and systemic immune dysfunction are major factors in the development of SBP [184]. These cascades of events promote bacterial translocation from the intestinal lumen to the mLNs, portal vein, and ascites. Eventually, under proper conditions, infection occurs [185]. A recent study [142] revealed that *Escherichia coli* and *Proteus mirabilis* (*P. mirabilis*) in ascites from patients with SBP caused a significant reduction in intercellular junctions in a dose‐ and time‐dependent manner. This effect is enhanced by the direct interaction of live bacteria with epithelial cells. Increased proteasomal ubiquitination mediates occludin degradation, and novel bacterial protease activity is essential for E‐cadherin cleavage. Therefore, blocking these two mechanisms may constitute a new therapeutic strategy to prevent the occurrence of SBP in patients with cirrhosis. Another study [186] revealed that with the progression of cirrhosis, IELs and lamina propria lymphocytes in the intestinal mucosa of rats develop a proinflammatory pattern of immune dysregulation. This disordered immune system is characterized by the expansion of activated lymphocytes, a switch to a T helper 1 (Th1) regulatory pattern, and a decrease in Th17 cells. In rats with cirrhosis and ascites, this state is associated with the disruption of epithelial junction proteins, loss of fecal albumin, and intestinal bacterial ectopia. Therefore, current research on the pathogenesis of SBP focuses mainly on intestinal tight junction proteins, with fewer studies focusing on GVB and immune barriers.

In terms of treatment, FXR agonists such as obeticholic acid can stabilize the GVB and reduce bacterial translocation through the portal venous pathway in patients with cirrhosis [110, 187]. Another study revealed that oral lactulose can increase the abundance of intestinal *Bifidobacteria*, produce high concentrations of acetate, acidify the intestinal lumen of humans and mice, reduce the growth of drug‐resistant bacteria (such as vancomycin‐resistant *Enterococcus faecium*), and ultimately reduce the incidence of SBP [188]. Therefore, future prevention and treatment strategies for SBP should move beyond traditional antibiotic treatment to adopt multitarget comprehensive interventions, such as regulating the intestinal flora, repairing the intestinal barrier and correcting immune imbalance, to fundamentally break the vicious cycle of bacterial translocation. Table 1 summarizes the characteristic changes in gut‒liver axis dysregulation in major liver disease types.

**TABLE 1:** Key alterations in the gut–liver axis associated with major types of human liver diseases.

Liver diseases	Changes in gut bacteria	Changes in intestinal metabolites	Alterations in the intestinal barrier	Effects on liver disease
MAFLD	*Bifidobacterium* ↓ [9] *Eubacterium rectale* ↓ [189] *Escherichia coli* ↑ [94] *Veillonellaceae* ↑ [95]	Indoles ↓ [190] Ethanol ↑ [94] Secondary BAs↑ [191] Phenylactetate ↑ [192]	Intestinal tight junction proteins ↓ [94] PV‐1↑ [97]	Aggravate MAFLD Inhibit GVB disruption prevents the development of MASH
ALD	*Bifidobacterium* ↓ [9] *Enterococcus faecalis* ↑ [193] *Enterobacteria* ↑ [194] *Akkermansia muciniphila* ↓ [124]	SCFAs ↓ [121] Indoles ↓ [122] Conjugated BA ↑ [195]	Mucus thickness ↓ Intestinal tight junction proteins ↓ Immune barrier ↓ [126]	Alcohol damages the intestinal barrier. Aggravate ALD
Liver cirrhosis	*Escherichia coli ↑* *Klebsiella pneumoniae ↑* *Eubacterium eligens* ↓ *Eubacterium rectale* ↓ *Faecalibacterium prausnitzii* ↓ [139]	LPS ↑ [140] Secondary/primary BA ratios ↓ [196]	Mucus thickness ↓ [110, 142] SIgA [144] Intestinal tight junction proteins ↓ [110, 142] PV‐1 ↑ [97]	Allows PAMPs to translocate into the circulation Increased systemic inflammation and infection in patients with cirrhosis
Liver cancer	*Enterococcus faecalis* ↑ [9, 169] *Klebsiella* ↑ [154] *Haemophilus ↑* *Akkermansia ↓* *Ruminococcus* ↓ [197]	Butyrate ↓ [173] Secondary BA↑ [172] LPS ↑ [198]	Intestinal tight junction proteins ↓ PV‐1 ↑ [178]	Bacteria and metabolites disrupt the intestinal barrier, regulate liver immunity, and promote inflammation and carcinogenesis.
SBP	*Escherichia coli* ↑ [142] *Bifidobacterium* ↓ [188]	Acetate ↓ Taurocholic acid ↑ [188]	Mucus thickness ↓ Intestinal tight junction proteins ↓ [142]	Bacteria destroy the intestinal barrier and cause infections such as SBP.

## 4 | Therapeutic Targets and Interventions Targeting the Gut‒Liver Axis

Core treatment strategies targeting the gut‒liver axis for liver disease include restoring barrier function, correcting bacterial imbalance, regulating bacterial metabolism, blocking harmful signals, and restoring beneficial signals (Table 2).

**TABLE 2:** Summary of gut–liver axis targeted therapy strategies.

Intervention strategies	Representative methods	Mechanisms	Applicable liver diseases
Probiotics	*Bifidobacterium pseudolongum* [57]	Acetate activates GPR43	HCC
Antibiotics	Rifaximin [199]	Reduce gut oralization and promote intestinal barrier repair	Cirrhosis
Fecal microbiota transplantation (FMT)	FMT enema [200]	Restore gut barrier function and and upregulate SCFAs biosynthesis	ALD
FXR agonist	Obeticholic acid [112]	Inhibit liver inflammation and fibrosis	MAFLD
Improve intestinal barrier function	B‐94 and BB‐2516 [142]	Inhibit bacterial protease activity	SBP

### 4.1 Targeting the Intestinal Barrier

Strengthening intercellular junctions could inhibit inflammation and reduce liver disease. ReFerm is a food product made from oatmeal fermented with *Lactobacillus plantarum* DSM 9843 that is rich in microbial metabolites. ReFerm has previously been shown to reduce intestinal barrier permeability and effectively treat active ulcerative colitis [201]. A single‐center randomized controlled trial that enrolled 56 patients with advanced compensated ALD demonstrated that compared with standard nutritional support, 24 weeks of ReFerm treatment reduced intestinal barrier dysfunction, promoted liver regeneration, and reduced liver fibrosis, potentially halting the progression of ALD [202].

*Escherichia coli* and *Proteus mirabilis* in the ascites of SBP patients can induce intestinal proteasome ubiquitination to increase the degradation of occludin, and a new bacterial protease can cleave E‐cadherin. This protease activity can be partially blocked by matrix metalloproteinase inhibitors such as B‐94 and BB‐2516 [142]. Protease inhibition, which was originally established for cancer treatment, has evolved from a strategy targeting a wide range of proteases to a strategy targeting specific proteases and has expanded to the fields of inflammatory bowel disease and functional gastrointestinal disorders [203]. Targeting bacterial proteases provides a new option for anti‐infection treatment while protecting intestinal mucosal barrier function and preventing the progressive deterioration of liver disease.

### 4.2 | Targeting the Intestinal Flora

Current strategies that involve targeting the intestinal flora to treat liver disease mainly include the use of probiotics, prebiotics, antibiotics, FMT, and phages. *Lactobacillus* and *Bifidobacterium* are the two most commonly used probiotics, and the combined use of multiple probiotics can achieve more significant therapeutic effects. VSL#3 contains four lactic acid bacteria, three bifidobacteria and *Streptococcus thermophilus* DSM24731. A randomized clinical study conducted in 2014 included 130 patients with cirrhosis and revealed that the continuous use of VSL#3 for 6 months significantly reduced the risk of hospitalization due to complications in patients with cirrhosis, and the Child–Pugh and end‐stage liver disease model scores were also significantly reduced [204]. *Bifidobacterium pseudolongum* was the most consumed bacterium in MAFLD–HCC mice. In a mouse model, oral administration of *Bifidobacterium pseudolongum* significantly inhibited the formation of MAFLD–HCC, indicating that this bacterium has certain application prospects in MAFLD–HCC [57].

Engineered bacteria represent another research frontier for targeted microbial therapy. Researchers have genetically engineered *Escherichia coli* Nissle 1917 (EcN) to create a strain containing three genes: IsmA, BSH, and BCoAT. This strain, combined with an oleic acid‐inducible system, can effectively reduce serum cholesterol levels in mice fed a HFD, improve intestinal permeability, and repair liver damage [205]. In addition, l‐arginine in tumors is a key factor in determining the efficiency of antitumor T cell responses. Increasing the usually low concentration of l‐arginine in tumors through engineered bacteria can significantly enhance the antitumor response to immune checkpoint inhibitors. The results show that engineered microbial therapy can enhance the effect of immunotherapy by regulating the TME [206].

Prebiotics are defined as nondigestible food substances that resist breakdown by host digestive enzymes but can be selectively metabolized by beneficial gut microbiota. By modulating the microbial composition or generating bioactive metabolites, they confer health benefits on the host. Common prebiotics include fructooligosaccharides, inulin, resistant starch, and galactooligosaccharides (GOS). Compared with insoluble dietary fiber, inulin can more effectively inhibit liver steatosis, necrotic inflammation, balloon‐like disease and fibrosis in mice. The commensal *Parabacteroides distasonis* uses inulin to produce pentadecanoic acid, upregulating the expression of intestinal tight junction proteins in the MASH model and thereby reducing the levels of serum LPSs and liver proinflammatory cytokines [102]. A 4‐month randomized controlled clinical trial conducted on 196 MAFLD patients revealed that the abundance of *Bacteroides stercoris* in patients in the resistant starch group significantly decreased, the serum branched chain amino acid concentration increased, and ultimately, the absolute triglyceride content in the liver decreased, suggesting that this may constitute a new strategy for the treatment of MAFLD [207].

Oral intestinal nonabsorbable antibacterial drugs can inhibit intestinal bacterial overgrowth, reduce intestinal NH3 production and absorption, and promote the growth of beneficial bacteria such as *Bifidobacteria* and *Lactobacilli*. Rifaximin (RFX) is an oral, broad‐spectrum, bactericidal antibiotic with a low gastrointestinal absorption rate. It is currently used to treat recurrent hepatic encephalopathy. A randomized controlled trial involving 136 patients with ALD revealed that regular treatment with rifaximin‐α for 18 months could reduce the progression of liver fibrosis, but further confirmation is needed through a multicenter phase 3 trial [208]. A recent study [209] of patients with severe cirrhosis and ascites revealed that rifaximin, when used as a primary prevention therapy for SBP, did not improve survival or reduce the incidence of liver complications (such as SBP, gastrointestinal bleeding, hepatic encephalopathy, or hepatorenal syndrome) after 12 months. However, in the subgroup of patients who strictly adhered to rifaximin therapy, the incidence of cirrhosis complications was reduced. Experiments in male C57BL/6J mice revealed that the antibiotic combination (ampicillin/vancomycin/metronidazole/gentamicin) could slow the progression of MAFLD without affecting the integrity of the intestinal barrier [210]. Fecal microbiota from MASH patients were transplanted into germ‐free C57BL/6 mice, which were then fed a Western diet (WD) for 20 weeks. Antifungal treatment with amphotericin B can reduce hepatic triglyceride and cholesterol concentrations and alleviate liver damage [189].

FMT is a medical technique that involves transplanting the functional flora from the feces of healthy donors into the intestines of patients to restore the balance of their intestinal microecology. After 8 weeks of FMT, the imbalance of intestinal flora in mice fed a HFD improved, and the concentration of butyrate in the contents and the level of the intestinal tight junction protein ZO‐1 increased. Ultimately, endotoxemia is alleviated, and fatty liver inflammation is effectively reduced [211]. Clinical studies have shown that FMT is safe and well tolerated and significantly reduces the recurrence rate of hepatic encephalopathy, regardless of the route of administration (oral or enema), number of doses (one to three times), or donor type (vegan or omnivore) [212]. However, given the potential risk of transmitting uncharacterized or pathogenic bacteria, it seems safer to use specific probiotics and their characterized products instead of FMT.

Bacteriophages (also known as phages) are a type of virus that specifically infects bacteria and are widely present in nature (such as soil, water, and animal intestines). The emergence of drug‐resistant bacteria has brought renewed interest in bacteriophages for the treatment of infectious diseases. Additionally, because certain bacteria play important roles in liver diseases, the selective elimination of these pathogenic organisms to restore the symbiotic balance is a promising approach [213]. The presence of cytolytic *Enterococcus faecalis* correlated with liver disease severity and mortality in patients with alcoholic hepatitis, and phages targeting cytolytic *Enterococcus faecalis* reduced cytolysin in the liver and abolished ethanol‐induced liver disease in humanized mice implanted with feces from patients with alcoholic hepatitis [193]. Similarly, high alcohol‐producing *Klebsiella pneumoniae* (HiAlc Kpn) may be a cause of MAFLD. Experiments in mice have shown that treatment with HiAlc Kpn‐specific phages can alleviate steatohepatitis, including improving liver dysfunction and reducing the expression of cytokines and lipogenic genes [214]. Phages are highly specific and do not destroy beneficial commensal bacteria in the intestine. This is in stark contrast to the “sweep away” effect of broad‐spectrum antibiotics and can better maintain the balance of the microecology. However, the use of phages to target intestinal bacteria for the treatment of liver disease is still in its early stages, and no large‐scale clinical studies have been reported. It can only lyse limited strains, and bacteria can develop different resistance mechanisms to escape phage invasion, greatly reducing the efficacy of phage therapy. To control phage resistance, multiple phages can be combined or used in conjunction with antibiotics to prevent the emergence of phage‐resistant bacteria [215].

### 4.3 | Targeting Microbial Metabolites and Signaling Pathways

The BA metabolic pathway is the main pathway for cholesterol degradation in the liver. It is not only involved in lipid digestion and absorption but also regulates energy metabolism and signal transduction. A group of gut microbiota‐modified BAs, namely, hyodeoxycholic acid (HDCA), is negatively correlated with the presence and severity of MAFLD. HDCA alleviates MAFLD in multiple mouse models by inhibiting intestinal FXR and upregulating hepatic CYP7B1. In addition, HDCA significantly increased the abundance of *Parabacteroides distasonis*, a probiotic that enhances lipid catabolism through fatty acid‐hepatic peroxisome proliferator‐activated receptor α signaling, which in turn upregulates hepatic FXR [216]. Another study revealed the previously unreported molecule 3‐succinylcholic acid (3‐sucCA), which was negatively correlated with liver damage in patients with MAFLD confirmed by liver biopsy. In both in vitro and in vivo experiments, *Bacteroides uniformis* strains effectively promoted the synthesis of 3‐sucCA. Further studies have shown that 3‐sucCA is an intestinal lumen‐restricted metabolite that can alleviate MASH by promoting the growth of *Akkermansia muciniphila* [217]. In addition to serving as energy sources for intestinal cells, SCFAs can also act on signaling pathways to affect liver metabolism. The acetate and valeric acid produced by probiotics inhibit the development of MAFLD‐related HCC by binding to the hepatocyte GPR43 receptor and downregulating the activity of the IL‐6–JAK–STAT3 and Rho–GTPase pathways, respectively [57, 58]. This first‐in‐human study revealed that delivering propionate directly to the colon significantly increased the release of GLP‐1 and reduced energy intake. Propionate treatment for 24 weeks significantly reduced weight gain, intra‐abdominal adipose tissue distribution, and intrahepatocyte lipid content in patients with MAFLD [218].

TLR4 mediates endotoxin‐induced tissue damage in liver failure and cirrhosis, with upregulated liver TLR4 expression and increased blood TLR4 ligands in patients with cirrhosis. The inhibition of TLR4 signaling using a TLR4 inhibitor (TAK‐242) improved organ damage and systemic inflammation in a rat liver failure model [219]. Further mouse models have demonstrated that the combination of granulocyte colony‐stimulating factor G‐CSF (which can promote liver regeneration) and TAK‐242 (which can inhibit receptors that play a key role in inflammation) is significantly better than either of the other drugs alone and can effectively treat acute‐on‐chronic liver failure [220].

### 4.4 Dietary and Lifestyle Interventions

A high‐fat, low‐fiber WD can induce microbiome dysbiosis, characterized by reduced diversity and metabolic breadth, which can increase the risk of cardiovascular, metabolic, and autoimmune diseases [221]. A recent study [222] analyzed the recovery of intestinal microbiota in mice fed regular chow and a WD after antibiotic treatment and revealed that only mice fed regular chow experienced rapid recovery. Compared with FMT, specific diets may provide safer, more effective and less invasive alternatives for addressing dysbiosis. The Mediterranean diet uses fruits, fresh vegetables, minimally processed whole grains, legumes, and fish in the greatest proportions, and omega‐3 fatty acids (such as olive oil and nuts) are the main source of fat, supplemented by relatively small amounts of dairy products, fructose, and meat. This study revealed that for every one standard deviation increase in the Mediterranean diet score (a measure of Mediterranean diet intake), there was a 26% lower incidence of MAFLD. Furthermore, an increase in the Mediterranean diet score was associated with a decrease in the accumulation and severity of fat in the liver [223]. A fasting‐mimicking diet (FMD) simulates the physiological response of the body in a fasting state by limiting calories, low carbohydrates, and low protein, resulting in similar health benefits to fasting while avoiding the discomfort and potential risks of complete fasting. After the subjects completed three FMD cycles, their insulin resistance and other prediabetes indicators decreased, and their liver fat content (measured by magnetic resonance imaging) significantly decreased [224].

The American College of Gastroenterology recommends that patients with MAFLD engage in regular physical activity each week, with the goal of 150–300 min of moderate‐intensity exercise or 75–150 min of vigorous‐intensity aerobic exercise [225]. Physical activity (independent of weight loss) can increase liver fat content and peripheral sensitivity to insulin, reduce de novo hepatic lipogenesis, and reduce the delivery of free fatty acids to the liver [226]. A prospective cohort study in the United States included 125,264 participants (52.82% of whom had a BMI > 25) and reported that the risk of liver‐related death increased monotonically with increasing BMI (*p* < 0.0001), whereas walking at a moderate pace for more than 3 h per week could prevent 25% of liver‐related deaths (95% CI 0.12–0.38) [227]. Exercise training can reverse intestinal flora dysbiosis in patients with MASH, and both 16S rRNA and metatranscriptomic data show that the intestinal flora and functional diversity are relatively high. Second, exercise training can increase the prevalence of *Parabacteroides distasonis*, and its low abundance can lead to the occurrence of MASH. The enrichment of *Parabacteroides distasonis* establishes a variety of positive symbiotic relationships with intestinal commensal bacteria to jointly maintain intestinal health [228, 229]. Finally, we summarized recent clinical studies on liver disease treatment based on the gut‒liver axis (Table 3).

**TABLE 3:** Summary of clinical studies on liver disease treatment based on the gut–liver axis.

Intervention	Registration number	Objectives	Populations and groups	Preliminary findings	References
**MAFLD**					
Resistant starch versus placebo for 4 months	ChiCTR‐IOR‐15007519	To investigate the effects of resistant starch on MAFLD	196 patients with MAFLD, randomized 1:1	The resistant starch group had an increase in serum BCAAs concentrations, which ultimately reduced the absolute value of triglyceride content in the liver.	[207]
A. muciniphila versus placebo for 3 months	NCT02637115	To investigate the effects of *A. muciniphila* on safety, tolerability, and metabolic parameters in obese patients	32 overweight/obese insulin‐resistant volunteers, randomized 1:1:1	3 months of supplementation with *A. muciniphila* reduced levels of blood markers associated with liver dysfunction and inflammation, while the overall gut microbiota structure was not affected.	[230]
Synbiotic agents versus placebo for 14 months	NCT01680640	To investigate whether a synbiotic combination affects MAFLD biomarkers and the fecal microbiota	104 patients with MAFLD, randomized 1:1	Taking a synbiotic combination (probiotics and prebiotics) for 1 year altered the fecal microbiota but did not reduce liver fat content or markers of liver fibrosis.	[231]
**ALD**					
Oral *Lactobacillus rhamnosus* GG (LGG) versus placebo for 6 months	NCT01922895	To evaluate the safety and efficacy of 6 months of LGG supplementation in patients with ALD	46 patients with ALD, randomized 1:1	After 1 month of LGG treatment, liver damage was significantly reduced. Six months of LGG treatment reduced heavy drinking levels to social or abstinence levels.	[129]
Rifaximin versus placebo for 18 months	EudraCT 2014‐001856‐51	To evaluate the efficacy of rifaximin‐α in patients with biopsy‐proven ALD	136 patients with ALD, randomized 1:1	Rifaximin can slow down the progression of liver fibrosis without obvious adverse reactions.	[208]
Prebiotic (inulin) versus placebo for 17 days	NCT03803709	To investigate the effects of inulin on liver parameters in subjects with ALD	50 patients with ALD, randomized 1:1	Although 17 days of inulin supplementation induced specific changes in the intestinal flora, it did not alleviate liver damage in patients with ALD.	[232]
ReFerm versus standard nutritional support for 24 weeks	NCT03863730	To investigate the efficacy of ReFerm on liver fibrosis in patients with ALD	56 patients with ALD, randomized 1:1	ReFermcan alleviate intestinal barrier dysfunction, promote liver regeneration and reduce liver fibrosis.	[202]
**Cirrhosis**					
Probiotic VSL#3 versus placebo for 6 months	NCT01110447	To assess whether probiotics affect outcomes in patients with cirrhosis and HE	130 cirrhosis patients who recovered from HE last month, randomized 1:1	Daily intake of VSL#3 significantly reduces the risk of hospitalization for HE and the severity of liver function in patients with cirrhosis.	[204]
FMT versus placebo for 6 months	NCT03796598	To evaluate the effects of different routes and doses of FMT on the prevention of HE in patients with cirrhosis	Patients with cirrhosis and HE on lactulose and rifaximin, randomized 1:1:1:1	FMT is safe, and no FMT‐related adverse events have been reported regardless of dose, route of administration, or donor type.	[212]
Yaq‐001 (engineered carbon bead adsorbent) versus placebo for 3 months	NCT03202498	To explore the safety and tolerability of Yaq‐001 in a clinical trial of cirrhosis	28 patients with cirrhosis, randomized 1:1	Yaq‐001 exhibited rapid adsorption kinetics for endotoxin, reduced intestinal permeability of organoids, and met its primary endpoints of safety and tolerability in clinical trials.	[233]
**HCC**					
Probiotic users versus placebo, retrospective study	/	To determine the potential association between probiotics and HCC	884 patients with hepatitis B‐related cirrhosis	The incidence of hepatocellular carcinoma was significantly lower in probiotic users than in nonusers (adjusted hazard ratio 0.70, *p* < 0.001).	[234]
Probiotic bacteria cocktail versus placebo during perioperative period	NCT05178524	To test whether *Bifidobacterium longum* can promote liver function recovery in patients with HCC after surgery	176 patients undergoing liver resection, randomized 1:1	Oral administration of a probiotic mixture containing *Bifidobacterium longum* reduced the rate of delayed recovery, shortened the length of hospital stay, and increased 1‐year overall survival.	[173]
**SBP**					
Rifaximin versus placebo for 12 months	NCT03069131	To evaluate whether rifaximin can provide primary prevention of SBP and improve outcomes in patients with cirrhosis and ascites	159 patients with severe cirrhosis and ascites, randomized 1:1	Rifaximin has no beneficial effect on survival or the incidence of cirrhosis complications, but improving patient compliance may reduce liver complications (including SBP).	[209]

## 5 Challenges, Future Perspectives, and Conclusions

As the core bridge connecting the intestine and the liver, the gut‒liver axis plays a central role in the occurrence and development of various liver diseases. It is essentially a highly complex, dynamic and bidirectionally interacting system. Current research has revealed the key mechanisms that drive the progression of liver disease, including intestinal barrier function disruption, dysbiosis, metabolic product disorders, and the activation/inhibition of abnormal signaling pathways. These findings provide a strong theoretical basis for intervention targeting the gut‒liver axis, making it a highly promising new therapeutic strategy and drug development hot spot.

**FIGURE 3:**
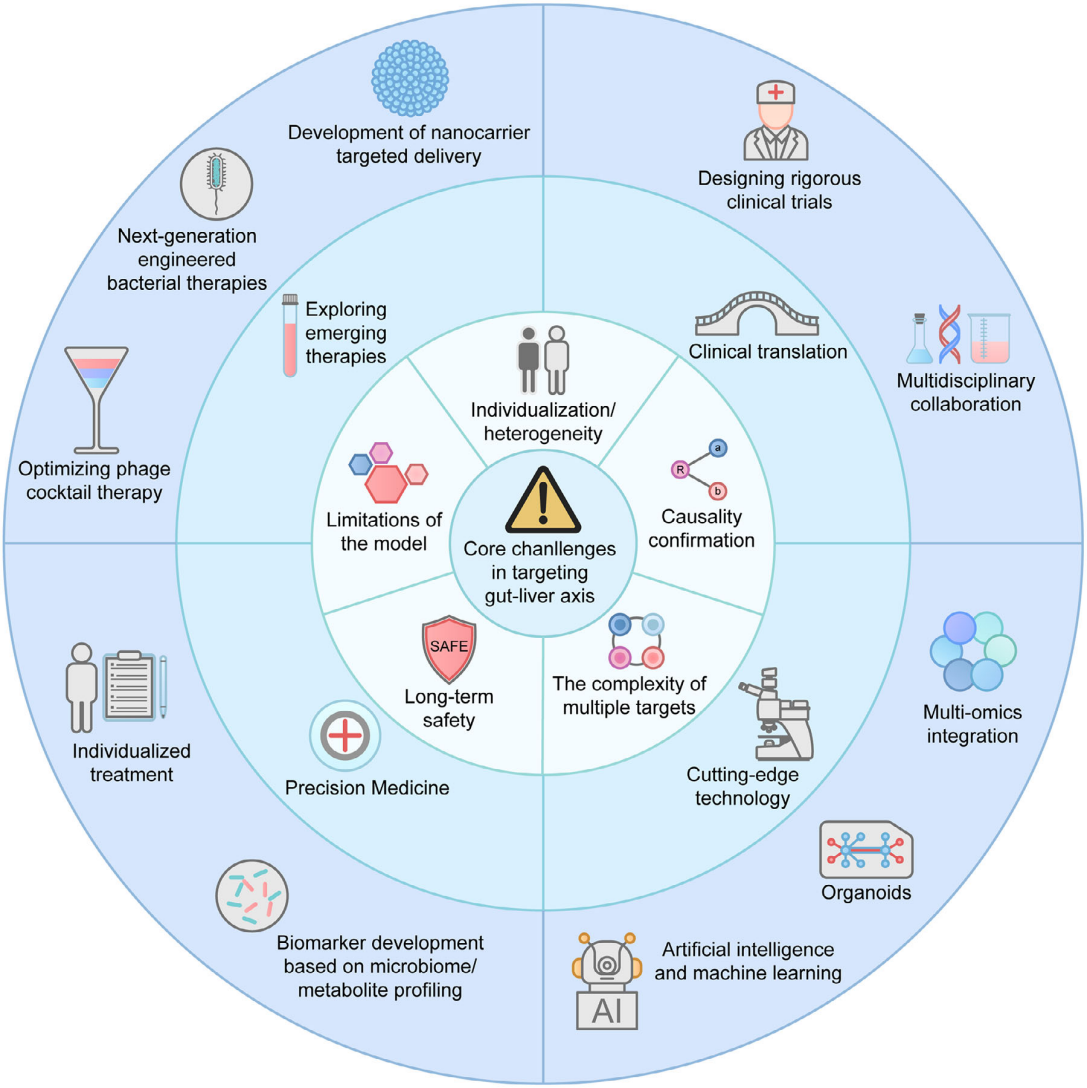
Conceptual diagram of future directions and challenges in gut–liver axis research. As a bidirectional interactive system connecting the intestine and liver, the gut–liver axis plays a central role in the development and progression of liver disease and has emerged as a promising therapeutic target. However, this field still faces challenges such as high individual variability, unclear causal mechanisms, low translational efficiency, and therapeutic safety. Future advances will require the utilization of cutting‐edge strategies such as multiomics technologies, personalized precision medicine, engineered bacteria, and nanodelivery, along with rigorous clinical trials to promote translational applications and ultimately achieve breakthroughs in liver disease treatment. Created with Adobe Illustrator.

However, this field still faces significant challenges (Figure 3). The highly individualized and dynamic changes in the intestinal flora make it difficult to develop universal therapies, scientific barriers to inferring causal relationships from correlations still exist, and the efficiency of converting animal model research results to human applications is low. In addition, the long‐term safety and regulatory framework of therapeutic interventions (such as FMT and specific probiotics) need to be improved, and the complexity of multitarget combination therapy also increases the difficulty of research and development.

Looking ahead, the key to overcoming these challenges and unleashing the therapeutic potential of the gut‒liver axis lies in multidisciplinary integration and technological innovation. Integrating multiomics methods such as metagenomics, metabolomics, and single‐cell technology will deepen our understanding of the underlying mechanisms of the axis. The development of personalized diagnostic markers and precision therapies based on the microbiota/metabolites is likewise important. Exploring cutting‐edge technologies such as engineered bacteria, phage cocktail therapy, and nanodelivery systems will expand treatment options, and designing rigorous clinical trials that are biomarker driven and stratified for treatment is essential. Although the road ahead is full of challenges, with in‐depth analysis of the gut‒liver axis mechanism and rapid technological advances, precision intervention strategies based on this axis are expected to lead to revolutionary treatment for patients with liver disease. Strengthening the close collaboration and transformation between basic research and clinical applications will be the core driving force to achieve this goal.

## Author Contributions

Zhiji Chen conceived, wrote, and edited the manuscript. Liao Siqi and Wu Suhua were responsible for the creation and improvement of figures and tables, literature search and analysis, and drafting important parts of the manuscript. An Zhang, Song He, and Zhi‐hang Zhou revised the manuscript. Siyuan Chen, Qin Tang, Li Zhou, Xiaoqin Li, Huiyi Hu, and Jiayao Xu provided significant assistance. All authors have read and approved the final manuscript.

## Conflicts of Interest

The authors declare no conflict of interest.

## Ethics Statement

The authors have nothing to report.

## Data Availability

The authors have nothing to report.
